# Local Ca^2+^ Entry Via Orai1 Regulates Plasma Membrane Recruitment of TRPC1 and Controls Cytosolic Ca^2+^ Signals Required for Specific Cell Functions

**DOI:** 10.1371/journal.pbio.1001025

**Published:** 2011-03-08

**Authors:** Kwong Tai Cheng, Xibao Liu, Hwei Ling Ong, William Swaim, Indu S. Ambudkar

**Affiliations:** Secretory Physiology Section, Molecular Physiology and Therapeutics Branch, NIDCR, NIH, Bethesda, Maryland, United States of America; University of Texas at Austin, United States of America

## Abstract

Store-operated Ca^2+^ entry (SOCE) has been associated with two types of channels: CRAC channels that require Orai1 and STIM1 and SOC channels that involve TRPC1, Orai1, and STIM1. While TRPC1 significantly contributes to SOCE and SOC channel activity, abrogation of Orai1 function eliminates SOCE and activation of TRPC1. The critical role of Orai1 in activation of TRPC1-SOC channels following Ca^2+^ store depletion has not yet been established. Herein we report that TRPC1 and Orai1 are components of distinct channels. We show that TRPC1/Orai1/STIM1-dependent I_SOC_, activated in response to Ca^2+^ store depletion, is composed of TRPC1/STIM1-mediated non-selective cation current and Orai1/STIM1-mediated I_CRAC_; the latter is detected when TRPC1 function is suppressed by expression of shTRPC1 or a STIM1 mutant that lacks TRPC1 gating, STIM1(^684^EE^685^). In addition to gating TRPC1 and Orai1, STIM1 mediates the recruitment and association of the channels within ER/PM junctional domains, a critical step in TRPC1 activation. Importantly, we show that Ca^2+^ entry via Orai1 triggers plasma membrane insertion of TRPC1, which is prevented by blocking SOCE with 1 µM Gd^3+^, removal of extracellular Ca^2+^, knockdown of Orai1, or expression of dominant negative mutant Orai1 lacking a functional pore, Orai1-E106Q. In cells expressing another pore mutant of Orai1, Orai1-E106D, TRPC1 trafficking is supported in Ca^2+^-containing, but not Ca^2+^-free, medium. Consistent with this, I_CRAC_ is activated in cells pretreated with thapsigargin in Ca^2+^-free medium while I_SOC_ is activated in cells pretreated in Ca^2+^-containing medium. Significantly, TRPC1 function is required for sustained K_Ca_ activity and contributes to NFκB activation while Orai1 is sufficient for NFAT activation. Together, these findings reveal an as-yet unidentified function for Orai1 that explains the critical requirement of the channel in the activation of TRPC1 following Ca^2+^ store depletion. We suggest that coordinated regulation of the surface expression of TRPC1 by Orai1 and gating by STIM1 provides a mechanism for rapidly modulating and maintaining SOCE-generated Ca^2+^ signals. By recruiting ion channels and other signaling pathways, Orai1 and STIM1 concertedly impact a variety of critical cell functions that are initiated by SOCE.

## Author Summary

Store-operated Ca^2+^ entry is present in all cell types and determines sustained cytosolic [Ca^2+^] increases that are critical for regulating a wide variety of physiological functions. This Ca^2+^ entry mechanism is activated in response to depletion of Ca^2+^ in the endoplasmic reticulum (ER). When ER [Ca^2+^] is decreased, the Ca^2+^-sensor protein STIM1 aggregates in the ER membrane and moves to regions in the periphery of the cells where it interacts with and activates two major types of channels that contribute to store-operated Ca^2+^ entry: CRAC and SOC. While gating of Orai1 by STIM1 is sufficient for CRAC channel activity, both Orai1 and transient receptor potential channel 1 (TRPC1) contribute to SOC channel function. The molecular composition of SOC channels and the critical role of Orai1 in activation of TRPC1 have not yet been established. In this study, we demonstrate that TRPC1 and Orai1 are components of distinct channels, both of which are regulated by STIM1. Importantly, we show that Orai1-mediated Ca^2+^ entry triggers plasma membrane insertion of TRPC1 which is then gated by STIM1. Ca^2+^ entry via functional TRPC1-STIM1 channels provides additional increase in cytosolic [Ca^2+^] that is required for regulation of specific cell functions such as K_Ca_ activation. Together, our findings elucidate the critical role of Orai1 in TRPC1 channel function. We suggest that the regulation of TRPC1 trafficking provides a mechanism for rapidly modulating cytosolic [Ca^2+^] following Ca^2+^ store depletion.

## Introduction

Store-operated Ca^2+^ entry (SOCE) is activated in response to a reduction of [Ca^2+^] in the ER. SOCE generates local and global [Ca^2+^]_i_ signals that regulate a wide variety of cellular functions [1],[2]. The first store-operated Ca^2+^ channel to be characterized, the Ca^2+^ release-activated Ca^2+^ (CRAC) channel, has a high selectivity for Ca^2+^ versus Na^+^ and displays a typical inwardly rectifying current-voltage relationship. CRAC channel accounts for the SOCE in lymphocytes and mast cells [3]–[6] and has recently been detected in some other cell types [7]–[9]. Key molecular components of the channel are STIM1 and Orai1. STIM1 is an ER Ca^2+^ binding protein that has been established as the primary regulator of SOCE [10]–[12]. In response to store depletion STIM1 oligomerizes and translocates to ER/PM junctional domains where it aggregates into puncta. The site of these aggregates is the location where STIM1 interacts with and activates channels involved in SOCE [13]–[15]. Orai1 is the pore-forming subunit of the CRAC channel [16]–[18]. Following store depletion, Orai1, which is localized diffusely in the plasma membrane in resting cells, is recruited by STIM1 into the puncta and gated by interaction with a C-terminal region of STIM1 [19],[20]. While expression of this STIM1-domain induces spontaneous CRAC channel activation in extra ER/PM junctional domains, the site of the STIM1 puncta represents the cellular location where endogenous SOCE is activated by store depletion [21].

Store depletion also leads to activation of relatively non-selective Ca^2+^-permeable cation channels, usually referred to as SOC channels, that have been associated with SOCE in several other cell types [2],[22]–[25]. Despite more than a decade of studies, the molecular components of these channels have not yet been established and their function and regulation remain somewhat controversial. TRPC channels have been proposed as molecular components of SOC channels. Data in this regard are strongest for TRPC1 [2],[26]–[34] although TRPC3 and TRPC4 also appear to contribute to SOCE in some cell types [23],[25],[35]–[38]. Numerous studies show that disruption of TRPC1 attenuates SOCE and SOCE-dependent cell function [23],[26]–[34]. We have previously provided extensive data to demonstrate that TRPC1 is a critical component of SOC channels and SOCE in the human salivary gland cell line, HSG [30],[39]–[42]. Further, salivary gland acinar cells from TRPC1−/− mice display reduced SOCE and SOC channel activity, which account for loss of sustained K_Ca_ activation and, consequently, salivary fluid secretion [29]. However, the role of TRPC1 in SOCE has been questioned based on the lack of function of heterogously expressed channels [43]. Further, some tissues from TRPC1−/− mice do not display any changes in SOCE [44],[45]. The strongest evidence for the regulation of TRPC1 following store depletion has been provided by data demonstrating that STIM1 interacts with and activates TRPC1-SOC channels in response to Ca^2+^ store depletion [39],[42],[46]. SOC channels are attenuated by knockdown of endogenous STIM1 and spontaneously activated by expression of the STIM1 mutant, D76ASTIM1 [42],[46]. An important study showed that TRPC1 is gated by electrostatic interaction between STIM1(^684^KK^685^) and TRPC1(^639^DD^640^) [47].

An intriguing finding is that STIM1 alone is not sufficient for activation of TRPC1-SOC channels following Ca^2+^ store depletion. Functional Orai1 is also required since knockdown of Orai1 or expression of functionally deficit Orai1 mutants prevents TRPC1 activation [39],[48]. We have shown earlier that store depletion leads to the recruitment of a TRPC1/STIM1/Orai1 complex that is associated with the activation of SOCE [39],[42]. Thus, while STIM1 is the primary protein involved in SOC channel gating, both TRPC1 and Orai1 appear to contribute to SOC channel activity. There has been much debate about the essential role of Orai1 in TRPC1-SOC channel function and more specifically regarding whether TRPC1 and Orai1 contribute to a single SOC channel pore or whether Orai1 is a regulatory subunit of SOC channels. In this study we have assessed the critical role of Orai1 in regulation of TRPC1 function following intracellular Ca^2+^ store depletion and determined the contributions of TRPC1 and Orai1 to SOCE. We report that TRPC1 and Orai1 constitute two distinct channels that contribute to SOCE in HSG cells. Suppression of TRPC1 function unmasks the underlying CRAC channel function. Further, in response to store depletion, STIM1 mediates association of Orai1 and TRPC1 within ER/PM junctional domains. Ca^2+^ entry via Orai1/STIM1-CRAC channel triggers plasma membrane insertion of TRPC1 and gating is achieved by interaction with STIM1(^684^KK^685^) residues. Remarkably, while both Orai1 and TRPC1 contribute to [Ca^2+^]_i_ increase following store depletion, they impact different cellular functions. Ca^2+^ entry mediated by TRPC1 is the primary regulator of K_Ca_ channel and partially contributes to NFκB activation while Orai1-mediated Ca^2+^ entry alone is sufficient for maximal NFAT activation and partial NFκB activation. Together these findings reveal the molecular events that determine activation of TRPC1 channels following store depletion. We suggest that local Ca^2+^ entry mediated by Orai1 determines plasma membrane insertion of TRPC1 while gating by STIM1 controls its activation. Thus, Orai1 and STIM1 not only determine Ca^2+^ signals generated by CRAC channels but by regulating TRPC1 channel activity rapidly modulate [Ca^2+^]_i_ and thus significantly impact various cell functions.

## Results

### TRPC1, Orai1, and STIM1 Contribute to SOCE

**Figure 1:**
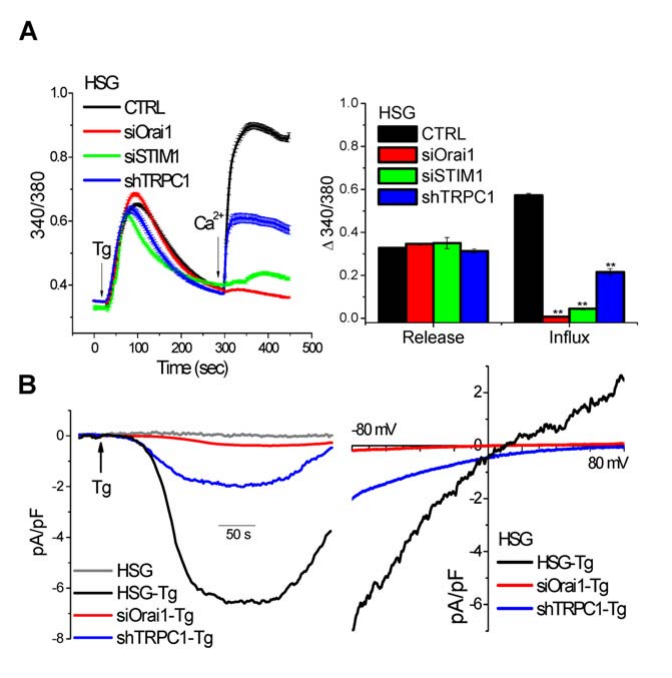
SOCE in HSG cells is determined by Orai1, TRPC1, and STIM1. (A) [Ca^2+^]_i_ measurements in fura-2 loaded HSG cells or (B) whole cell patch clamp recordings (see Material and Methods for details) in HSG cells treated as indicated to knock down either Orai1, STIM1, or TRPC1. Tg and Ca^2+^ were added as indicated. Right panel in (A) shows the average data for internal Ca^2+^ release (fluorescence increase prior to Ca^2+^ addition) and Ca^2+^ influx (fluorescence increase after Ca^2+^ addition; in each case resting fluorescence was subtracted from peak 340/380 ratio). ** indicate values that are significantly different (*p*<0.01) from the respective control value (black bars indicate values obtained in control cells). Data represent results from six different experiments and include at least 50–150 cells for each condition (see Figure S1 for additional data on TRPC1 knockdown under these conditions and the sensitivity of Tg-stimulated Ca^2+^ entry to Gd^3+^ and 2APB). (B) Left panel shows development of currents in cells following inclusion of Tg in normal external solution (arrow, traces depict currents measured at −80 mV; composition of the solutions used here and in the following experiments are provided in Materials and Methods). Right panel shows I–V relationship of the maximum currents recorded under the various conditions (note that currents are not detected without Tg stimulation of cells).

Compared to SOCE in control HSG cells (transfected with vector or scrambled siRNA; black traces in Figure 1), knockdown of endogenous Orai1, STIM1, or TRPC1 attenuated thapsigargin (Tg)-stimulated Ca^2+^ influx by >90%, >80%, or >60%, respectively (Figure 1A). These conditions did not significantly affect internal Ca^2+^ release. Western blots (Figure S1A) demonstrate the effectiveness of TRPC1 knockdown in these cells. Ca^2+^ entry induced by Tg treatment of HSG cells was blocked by 1 µM Gd^3+^ and 20 µM 2APB (Figure S1B). Further, expression of TRPC1, TRPC1+STIM1, Orai1+STIM1, or TRPC1+STIM1+Orai1 increased Tg-stimulated Ca^2+^ entry (Figure S1G), which was also blocked by 1 µM Gd^3+^ and 20 µM 2APB (Figure S1C–F). Together, these data are consistent with our previous studies [42] that Orai1, STIM1, and TRPC1 contribute to endogenous SOCE in HSG cells. Additionally, the contributions of TRPC1, STIM1, and Orai1 to SOCE were not dependent on the level of stimulation (Figure S2). The relative decrease in SOCE induced by individual knockdown of the three proteins was similar in cells stimulated with 100 µM carbachol (CCh, a maximal stimulatory concentration) or 1 µM CCh (submaximal stimulatory concentration).

The contribution of TRPC1 and Orai1 to SOCE in HSG cells was further examined by using whole cell patch clamp technique [2],[16],[17],[40] to record the current generated by intracellular Ca^2+^ store depletion (Figure 1B). Consistent with our previous findings, Tg stimulation of cells resulted in activation of I_SOC_ in HSG cells that is distinct from the typical I_CRAC_ currents measured in RBL cells and T lymphocytes [40]. We have previously reported [40] that I_SOC_ is a relatively Ca^2+^-selective cation current with E_rev_ around +20 mV and pCa^2+^/pNa^+^ = 40 (I_CRAC_ displays E_rev_>+60 mV and Ca^2+^/Na^+^ selectivity ≥400). Silencing of Orai1 expression blocked generation of I_SOC_ while knockdown of TRPC1 by shRNA significantly reduced the amplitude of the inward current but induced more pronounced loss of the outward current. Thus the residual current detected in 6/10 shTRPC1 treated cells was more inwardly rectifying, i.e. more like I_CRAC_ (Figure 1B, blue trace). These findings indicate the possibility that I_CRAC_ in HSG cells can be masked by the larger relatively non-selective TRPC1-mediated current that is activated under the same conditions. The extent of TRPC1 knockdown would then determine the detection I_CRAC_. In the present set of experiments, 40% of the cells displayed I_SOC_ or reduced I_SOC_. Our present data are somewhat contradictory to our previous finding that the residual current in Tg-stimulated submandibular gland acinar cells from TRPC1−/− mice was a much reduced transient current that was linear and did not display I_CRAC_-like properties (i.e. activation by low [2APB] or increase in DVF medium) [29]. We suggest that other TRPC channels or volume-regulated channels could account for the linear current. While further studies are required to determine the channel(s) involved in this residual current, our previous findings strongly demonstrate that TRPC1 contributes to SOCE and is critically required for salivary gland fluid secretion.

### Suppression of TRPC1 Function Unmasks I_CRAC_ in HSG Cells

**Figure 2:**
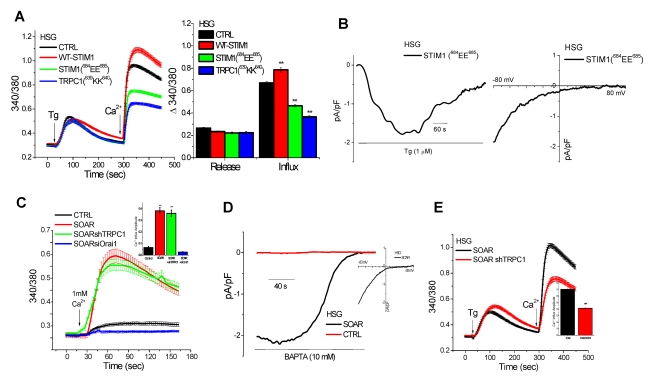
Endogenous I_CRAC_ is unmasked in HSG cells when TRPC1 function is suppressed by expression of STIM1(^684^EE^685^). HSG cells were transfected with plasmids encoding WT-STIM1, STIM1(^684^EE^685^), or TRPC1(^639^KK^640^). (A) Fura-2 fluorescence measurements in the cells indicated. Right panel shows average values for internal release and calcium influx (calculated as described for Figure 1). (B) Whole cell patch clamp measurements in HSG cells expressing STIM1(^684^EE^685^); left panel shows development of current with Tg in normal external solution (current recorded at −80 mV is shown) and right panel shows the I–V relationship of the current. Fura-2 (C, E) or patch clamp (D) measurements in HSG cells transfected with SOAR alone or together with shTRPC1 or siOrai1 (as indicated in each figure). Ca^2+^ was added to cells maintained in Ca^2+^-free medium (C, left panel) and average increase in fura-2 fluorescence, reflecting basal Ca^2+^ entry, is shown by the bar graphs in the inset (** indicates values that are significantly different, *p*<0.01, from other values but not from each other). (D) Spontaneously activated current recorded in SOAR-expressing HSG cells when 10 mM BAPTA was included in the pipette solution (see Materials and Methods for details). Inset shows the I_CRAC_-like I–V relationship of the current. (E) [Ca^2+^]_i_ measurements in Tg-treated HSG cells expressing SOAR or SOAR+shTRPC1. Inset shows average values obtained under these conditions. Note that basal Ca^2+^ entry in SOAR-expressing cells is similar to that seen in Tg-treated cells expressing SOAR+shTRPC1 (compare red traces in C and E). ** indicates values that are significantly different from control values (*p*<0.01, data obtained from at least five different experiments and include at least 50–60 cells for each condition).

The two C-terminal residues of STIM1(^684^KK^685^) mediate gating of TRPC1 via electrostatic interaction with TRPC1(^639^DD^640^) residues [47]. Consistent with this, expression of a STIM1 mutant that lacks ability to gate TRPC1, STIM1(^684^EE^685^), induced suppression of SOCE in HSG cells while expression of WT-STIM1 resulted in a small increase in function (Figure 2A). Expression of the TRPC1 mutant that cannot be gated by STIM1, TRPC1(^639^KK^640^), induced a similar suppression of endogenous SOCE (Figure 2A, blue trace). Further, TRPC1 was not activated by store depletion when expressed with STIM1(^684^EE^685^) in HEK293 cells (Figure S3A), but when STIM1 and TRPC1 mutants were expressed together (i.e. “charge swap” between the proteins) there was recovery of SOCE (Figure S3A). Importantly, STIM1(^684^EE^685^) stimulated Orai1 similar to WT-STIM1 (Figure S3B). A key finding of this study, shown in Figure 2B, is that expression of STIM1(^684^EE^685^) resulted in generation of I_CRAC_ in response to Tg-induced Ca^2+^ store depletion in >70% of HSG cells displaying currents. Together the data in Figures 1B and 2B suggest that I_SOC_ in HSG cells is composed of a small Orai1-mediated I_CRAC_ and a larger TRPC1-mediated non-selective current (note that we have not yet measured an isolated TRPC1+STIM1 current).

To conclusively demonstrate that endogenous Orai1 mediates I_CRAC_ in HSG cells we expressed the STIM1-Orai1-activating region (SOAR) [20]. A large increase in basal Ca^2+^ entry (Figure 2C) and spontaneous I_CRAC_ was seen in these cells (Figure 2D). SOAR-induced spontaneous SOCE was abolished by knockdown of endogenous Orai1 but was not affected by knockdown of endogenous TRPC1 (Figure 2C). In contrast, Tg-stimulated Ca^2+^ entry in SOAR-expressing cells was significantly reduced by knockdown of TRPC1 (Figure 2E, the residual Ca^2+^ entry reflects spontaneous Orai1-dependent Ca^2+^ influx). In aggregate, these data provide strong evidence that endogenous Orai1 mediates I_CRAC_ without any contribution from TRPC1 while SOCE and I_SOC_ display significant contribution from TRPC1. Importantly, the function of TRPC1 requires Orai1.

### Orai1-Mediated Ca^2+^ Entry Triggers Plasma Membrane Insertion of TRPC1 Channels

**Figure 3:**
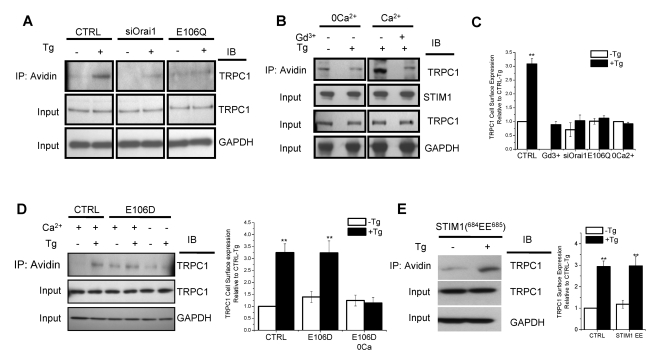
Orai1-mediated Ca^2+^ entry induces plasma membrane insertion of TRPC1 channels. Biotinylation of TRPC1 was determined in HSG cells (see Materials and Methods for details). (A) Detection of TRPC1 in the biotinylated fraction from control HSG cells and HSG cells treated with siOrai1 or expressing Orai1-E106Q in resting conditions or following treatment with Tg (see C for quantitation of the bands and average data from five individual experiments). (B) TRPC1 biotinylation in HSG cells treated with Tg either in the presence of Gd^3+^ or in Ca^2+^-free medium (see C for quantitation of data and average values obtained from four experiments). (C) Quantitation of TRPC1 surface expression shown in (A) and (B). ** indicates value significantly, *p*<0.01 from –Tg in control cells. All other values (unmarked) are represented relative to the –Tg condition in control cells and are not significantly different from each other. (D) TRPC1 biotinylation in HSG cells expressing Orai1-E106D with or without Tg treatment in either Ca^2+^-containing or nominally Ca^2+^-free medium (left panel). Average data (right panel, ** indicates values significantly, *p*<0.01, different from –Tg condition in controls, unmarked values are not different from each other). (E) TRPC1 biotinylation in HSG cells expressing STIM1(^684^EE^685^) with or without Tg treatment prior to surface biotinylation (left panel), average data (right panel, ** indicates values significantly, *p*<0.01, different from –Tg condition in controls; unmarked values are not different from each other).

To identify the mechanism involved in regulation of TRPC1-SOC channels we examined the effect of intracellular Ca^2+^ store depletion on the surface expression of TRPC1. In resting cells the surface expression of TRPC1 (i.e. in the biotinylated fraction) was relatively low. Tg treatment of cells (Figure 3A, left panel, total TRPC1 and GAPDH are shown in input) significantly enhanced (about 3-fold, Figure 3C) the insertion of TRPC1 into the plasma membrane. An important finding of this study (Figure 3A) is that Tg-stimulated increase in plasma membrane insertion of TRPC1 was dependent on Orai1. Decreasing Orai1 expression or compromising Orai1 function by expression of the dominant negative mutant Orai1-E106Q (Figure 3A, middle and right panels, respectively, see Figure 3C for average data) severely reduced Tg-stimulated surface expression of TRPC1 without significantly affecting the resting level of TRPC1. To examine whether Ca^2+^ entry was involved in TRPC1 trafficking, biotinylation of TRPC1 was assessed in cells stimulated with Tg in nominally Ca^2+^-free medium or in normal Ca^2+^-containing medium with 1 µM Gd^3+^. Both conditions blocked the increase in the surface expression of TRPC1 induced by Tg (Figure 3B and C). These effects on TRPC1 trafficking were not due to loss of TRPC1/STIM1/Orai1 clustering, which was not affected in cells expressing Orai1-E106Q [39] or in the absence of external Ca^2+^ (unpublished data). The role of Orai1-mediated Ca^2+^ entry was more directly assessed by using Orai1-E106D, an Orai1 mutant that is permeable to Ca^2+^ in Ca^2+^-containing medium, but unlike the wild type channel, it is permeable to Na^+^ in nominally Ca^2+^-free medium. Tg treatment of cells expressing this mutant induced surface expression of endogenous TRPC1 in Ca^2+^-containing medium but not in Ca^2+^-free medium (Figure 3D). Finally, trafficking of TRPC1 was examined in HSG cells expressing STIM1(^684^EE^685^), which display I_CRAC_ in response to Ca^2+^ store depletion (see Figure 2B). Although TRPC1 activation was suppressed in these cells, trafficking of the channel was not altered (Figure 3E). In aggregate these novel data suggest that Orai1-mediated Ca^2+^ influx is sufficient for plasma membrane insertion of TRPC1 but not activation; the latter depends on STIM1.

### STIM1 Mediates Co-clustering and Association of TRPC1 and Orai1

**Figure 4:**
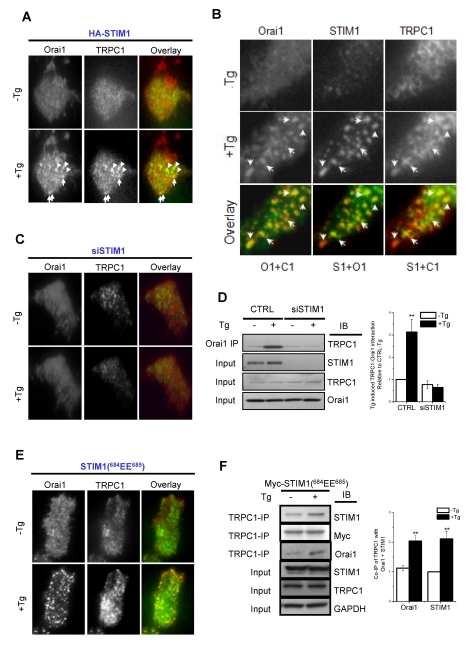
STIM1 determines clustering of TRPC1 and Orai1 in ER/PM junctional domains following Ca^2+^ store depletion. TIRFM was used to detect localization of TRPC1, STIM1, or Orai1 in HSG cells. (A) Localization of YFP-TRPC1 and CFP-Orai1 in HSG cells co-expressing HA-STIM1 before (−Tg) or 5 min after Ca^2+^-store depletion (+Tg). Left and central panels show localization of Orai1 and TRPC1 (shown by white arrows); right panels show co-localization of the proteins (Orai1-CFP, red, and YFP-TRPC1, green) with arrows showing examples of overlay (yellow) of TRPC1 and Orai1 punctae after Tg stimulation. (B) Similar experiment done with cells co-expressing Orai1-CFP, YFP-TRPC1, and mCherry-STIM1. To make the co-localization clear, in the overlay images, O1+C1 (TRPC1 is green, Orai1 is red), S1+O1 (Orai1 is green, STIM1 is red), and S1+C1 (STIM1 is red and TRPC1 is green). Arrows show examples of overlaying Orai1, TRPC1, and STIM1 punctae after Tg stimulation. (C) Localization of YFP-TRPC1 and Orai1-CFP in siSTIM1-treated HSG cells either with or without Tg (green signal indicates TRPC1 while red signal indicates Orai1). (D) Blot on left shows co-IP of endogenous TRPC1 and Orai1 in control and siSTIM1-treated cells (minus or plus Tg). Anti-Orai1 antibody was used for IP while anti-TRPC1 was used for IB. Inputs for TRPC1, STIM1, and Orai1 in each condition are shown in the lower three panels. The bar graph on the right shows quantitation of the blots from four similar experiments. ** indicates values significantly, *p*<0.01, different from controls, which are not different from each other. (E) TIRFM images of Orai1-CFP and YFP-TRPC1 in HSG cells expressing STIM1(^684^EE^685^). In the overlay, Orai1 is shown in red while TRPC1 is shown in green, with yellow indicating co-localization of the two proteins. (F) Tg-induced Co-IP of TRPC1, Orai1, and STIM1 in HSG cells expressing Myc-STIM1(^684^EE^685^) in resting conditions and after stimulation with Tg. Anti-TRPC1 antibody was used for IP and anti-Orai1; STIM1 or TRPC1 was used for IB, as indicated. Inputs and control blots are also shown. Quantitation of data from four similar experiments is shown by bar graph on the right, ** indicates values significant at *p*<0.01.

The mechanism involved in the clustering of TRPC1 with STIM1 and Orai1 was assessed by TIRFM. Ca^2+^ store depletion resulted in co-localization of YFP-TRPC1 and Orai1-CFP into puncta in the sub-plasma membrane region (Figure 4A, HA-STIM1 was co-expressed in these cells). Further, STIM1 co-clustered with both the channels following Tg stimulation of the cells (Figure 4B). As has been reported for Orai1, Orai1-TRPC1 clustering also required co-expression of STIM1 (unpublished data) and was not detected in cells when endogenous STIM1 expression was knocked down (Figure 4C). More significantly, co-IP of endogenous TRPC1 and Orai1 was abolished in cells treated with siSTIM1 (Figure 4D) but not in cells expressing STIM1(^685^EE^685^) (Figure 4E,F). TRPC1 clustering was not dependent on Orai1 since co-clustering of TRPC1 with STIM1 was unaffected by knockdown of Orai1 (Figure S4, compare data in A and B). Thus, STIM1 determines TRPC1 clustering in the sub-plasma membrane region following Ca^2+^ store depletion, and Orai1-mediated Ca^2+^ entry regulates its surface expression. Based on these findings we hypothesize that TRPC1 is present in recycling vesicles that traffic in and out of the plasma membrane region. Following store depletion when STIM1 clusters in ER/PM junctional domains, it interacts with TRPC1 possibly via the ERM domain [46] and increases the retention of TRPC1-containing vesicles. Concurrently, STIM1 also recruits Orai1 into the same regions, thus bringing the two channels in close proximity to each other. Ca^2+^ entry via Orai1 induces fusion of TRPC1-containing vesicles to the plasma membrane followed by gating of the channel by STIM1. Further studies are required to elucidate the mechanisms involved in trafficking and plasma membrane insertion of TRPC1.

### Local [Ca^2+^]_i_ Determines Plasma Membrane Insertion of TRPC1

**Figure 5:**
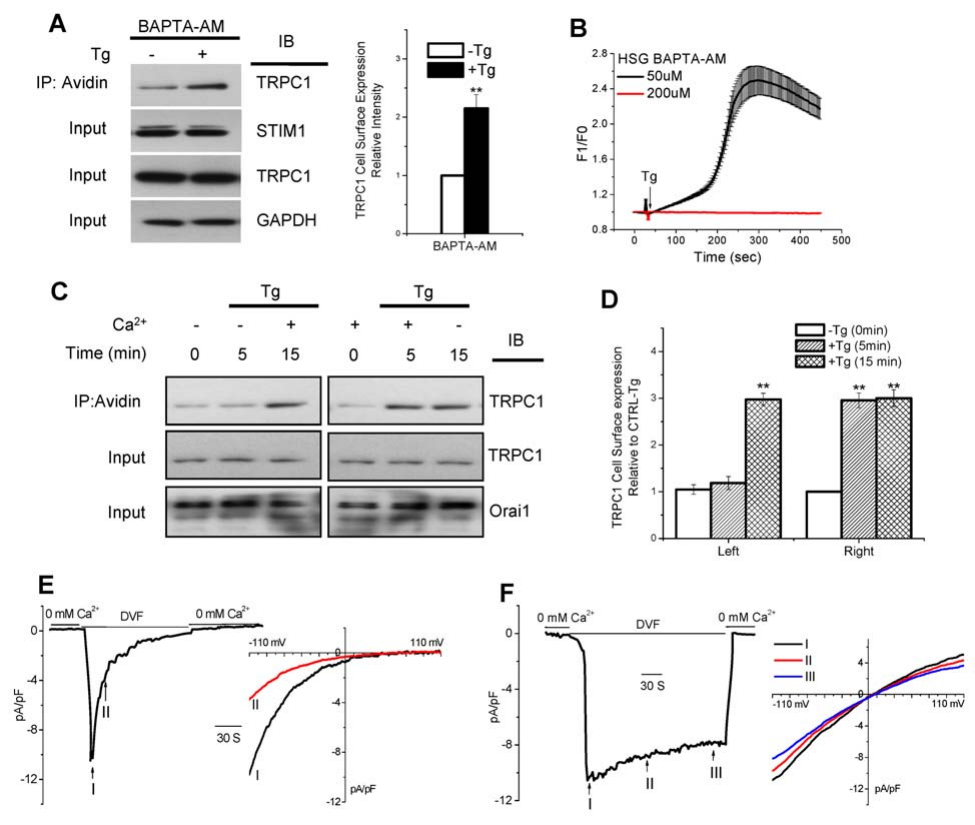
Local [Ca^2+^]_i_ determines plasma membrane insertion of TRPC1. (A) Tg treatment and biotinylation were carried out with HSG cells loaded with 200 µM BAPTA-AM (as described in Materials and Methods), and quantitation of TRPC1 in the fraction pulled down with avidin is shown on the right. The change induced by Tg is not significantly less than that seen in control cells in Figure 3. (B) Tg-stimulated increase in fura-2 fluorescence was measured in HSG cells loaded with either 50 µM or 200 µM BAPTA-AM. (C) Biotinylation of TRPC1 in cells treated under the conditions indicated in the figure. Levels of TRPC1 and Orai1 in the cell lysates are shown in the blot. (D) Quantitation of blots from four similar experiments as shown in (C) depict the increase in TRPC1 in the biotinylated fraction relative to control. ** indicates values significantly different from unmarked values but not from each other. (E, F) Effect of extracellular Ca^2+^ on detection of I_SOC_. HSG cells were either incubated for 10–15 min with Tg in nominally Ca^2+^-free medium (E) or Ca^2+^-containing medium (F). Cells were then patched in the Ca^2+^-free condition and then switched to DVF medium. In each case, current developments are shown by traces on the left and I–V curves of the current measured at the indicated time points are given on the right.

We next examined whether relatively global or local [Ca^2+^]_i_ increase regulates plasma membrane insertion of TRPC1. Figure 5A shows that loading HSG cells with 200 µM BAPTA-AM prior to Tg stimulation (details given in Methods) did not suppress trafficking of TRPC1 induced by Tg, although Tg-stimulated global [Ca^2+^]_i_ increase was completely suppressed (Figure 5B, compare red trace with black trace, which shows [Ca^2+^]_i_ increase in cells loaded with low [BAPTA-AM]). In addition, Tg-stimulated I_SOC_ was not altered by replacing EGTA in the pipette solution with 10 mM BAPTA (Figure S5B,C), although the latter condition completely suppressed K_Ca_ activation in Tg-stimulated cells (Figure S5C, right panel). TRPC4 and TRPC5 are directly activated by elevation of intracellular [Ca^2+^]_i_ [49], and a recent study demonstrated that Ca^2+^ entry mediated via Orai1 or other Ca^2+^ entry channels, including voltage-dependent channels, can directly enhance TRPC5 activity [50]. To determine whether [Ca^2+^]_i_ increase directly activates TRPC1, whole cell current measurement was done with [Ca^2+^] in the pipette solution clamped to 0.1 µM or 1 µM. No current was detected with 0.1 µM Ca^2+^ (unless Tg was included in the external medium, Figure S5A, black and blue traces), 1 µM Ca^2+^ (Figure S5A, red trace), or up to 5 µM Ca^2+^ (unpublished data). Note that 1 µM Ca^2+^ induces >90% activation of TRPC4 and TRPC5 [49],[50]. These data also rule out possible contribution of other Ca^2+^-dependent cation channels to SOCE [51].

In aggregate, these data suggest that local Ca^2+^ entry via Orai1 determines plasma membrane insertion of TRPC1 and that [Ca^2+^]_i_ elevation due to intracellular Ca^2+^ release is insufficient for triggering TRPC1 insertion. Further when cells were treated with Tg in a Ca^2+^ free medium for 5 min, there was no increase in TRPC1 expression in the plasma membrane until Ca^2+^ was added to the external solution (Figure 5C, right panel). As shown above, when cells were stimulated with Tg in a Ca^2+^-containing medium (Figure 5C, left panel), TRPC1 insertion in the plasma membrane was enhanced. Surprisingly, subsequent removal of Ca^2+^ from the external solution (for 10 min) did not change the level of TRPC1 in the surface membrane. Functional consequences of these treatments are shown in Figure 5E–F. In this experiment, HSG cells were treated with Tg in Ca^2+^-free medium prior to whole cell current measurements in DVF medium. Typical inwardly rectifying I_CRAC_ with rapid inactivation was detected in these cells (Figure 5E), consistent with the lack of TRPC1 insertion in the plasma membrane under these conditions. However, when pre-treatment was done in Ca^2+^-containing medium, I_SOC_ was detected in the DVF medium (Figure 5F). Note that the I_SOC_ in DVF was relatively sustained, again consistent with the stable biotinylation of TRPC1. In aggregate, the findings presented above suggest that Orai1-mediated Ca^2+^ entry triggers insertion of TRPC1 in the plasma membrane, followed by activation of the channel by STIM1. Thus while channel insertion into the plasma membrane appears to depend on local increases in [Ca^2+^]_i_, TRPC1 internalization does not strictly depend on a decrease in [Ca^2+^]_i_. Further studies will be required to determine the exact molecular mechanisms involved in internalization of TRPC1.

### Contribution of TRPC1 to SOCE-Regulated Cell Function

**Figure 6:**
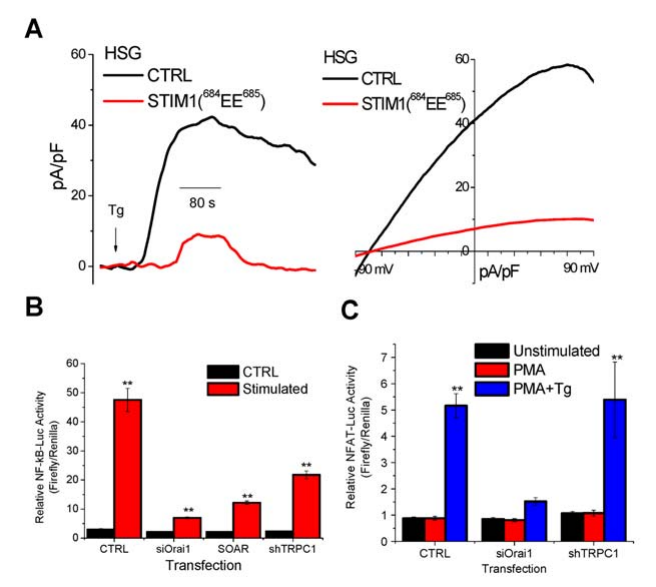
TRPC1-mediated Ca^2+^ entry regulates specific cellular functions. (A) Tg-induced activation of K_Ca_ channel in control HSG cells and cells expressing STIM1(^684^EE^685^) (note that these cells display I_CRAC_ when stimulated with Tg). Left panel shows development of the current at 0 mV and right panel shows the I–V relationship of the current. (B) Measurement of NFκB activation in HSG cells (control, +siOrai1, +SOAR, +shTRPC1). Conditions used for the experiments are shown in the figure and described in detail in Materials and Methods. (C) NFAT activation in HSG cells (control, +siOrai, or +shTRPC1).

The data presented above demonstrate that two STIM1-gated channels, Orai1 and TRPC1, are activated in response to internal Ca^2+^ store depletion in HSG cells. To establish the relative contributions of these channels in SOCE-mediated Ca^2+^ signaling, we examined three SOCE-activated mechanisms: K_Ca_ channel, NFκB, and NFAT. Figure 6A demonstrates that expression of STIM1(^684^EE^685^) in HSG cells induced a slow, much diminished (>80% reduction), and transiently activated K_Ca_ current compared to that in control cells. As shown above (Figure 2B), only CRAC channel activation was seen in cells expressing this STIM1 mutant. Thus, Orai1-mediated Ca^2+^ entry does not appear to be sufficient for activation of K_Ca_ activity following Tg stimulation. Further, NFκB activation (Figure 6B) was significantly decreased by the knockdown of TRPC1 expression, and predictably knockdown of Orai1 induced an even greater loss of activity. Significantly, expression of SOAR did not lead to much activation of NFκB. Remarkably, TRPC1 had minimal contribution to the regulation of NFAT since knockdown of Orai1 but not TRPC1 suppressed NFAT activation (Figure 6C). Thus, Orai1-mediated Ca^2+^ entry is sufficient for regulation of NFAT and for partial stimulation of NFκB, but not for K_Ca_ activation. In contrast, TRPC1-mediated Ca^2+^ entry regulates K_Ca_ channel activity and contributes to NFκB signaling, but not NFAT activation.

Similar to the findings in HSG cells, K_Ca_ activity was severely reduced in acinar cells from submandibular glands of TRPC1−/− mice, which could account for loss of salivary fluid secretion in these animals [29]. While our current findings suggest that Orai1+STIM1 dependent regulation of TRPC1 would be very critical for regulating salivary gland function, functional interaction between these proteins will depend on their precise localization within acinar cells, as is required in HSG cells (Figure 4B). We have previously reported that TRPC1 is localized in the basal and lateral regions of submandibular gland acinar cells [29],[52] and that TRPC1 and STIM1 co-IP following stimulation of acini by either Tg or CCh [53]. To determine possible physiological relevance of the present findings, we examined the localization of TRPC1, Orai1, and STIM1 in submandibular glands excised from resting and pilocarpine-stimulated mice (tissue was fixed in vivo in mice following pilocarpine injection and after an increase in saliva secretion was detected). In the samples from unstimulated mice, endogenous Orai1 was prominantly detected in the apical and lateral regions of submandibular gland acini (Figure S6A, upper panels, green signal, Orai1 signal shown by white arrows), co-localization of Orai1 with the luminal membrane protein AQP5 is also shown (red signal, right panel). STIM1 showed diffused localization within the acinar cells from unstimulated mice (Figure S6B, red signal, upper panel). Consistent with our previous findings, diffuse localization of TRPC1 was detected in the basal and lateral regions (green signal, upper panels, the same sections were labeled for STIM1 and TRPC1). In samples obtained from stimulated mice, Orai1 and AQP5 localization did not change (Figure S6A, lower panels). However, a dramatic translocation of TRPC1 and STIM1 to the basal and lateral membrane regions was seen with relative decrease in intracellular staining (Figure S6B, lower panels, see white arrows). Thus stimulation induces co-localization of STIM1, Orai1, and TRPC1 in the lateral membrane region of cells. While further studies are required to determine whether sufficient Orai1 is present in the basolateral membrane to regulate TRPC1, our data strongly suggest that regulation of TRPC1 by STIM1 and Orai1 is feasible within the lateral membrane region of salivary gland acinar cells. Our findings are generally consistent with the strong co-localization of Orai1 and STIM1 in the lateral membrane region of stimulated pancreatic acinar cells [54]. STIM1 was also localized in the basal membrane and co-localized with heterologously expressed, but not endogenous, Orai1, in these cells. This study suggested that localization of Orai1 and STIM1 in the lateral membrane was consistent with the proposed site of Ca^2+^ entry in exocrine acinar cells [55]–[57].

## Discussion

The findings described herein address several important and as-yet unresolved questions regarding the molecular components of TRPC1-SOC channel, the mechanism involved in regulation of the channel in response to store depletion, and its contribution to SOCE. We report that the previously described I_SOC_ [39],[40],[58], which is stimulated by store depletion and dependent on TRPC1, STIM1, and Orai1, is a sum of Orai1/STIM1-mediated I_CRAC_ and TRPC1/STIM1-mediated non-selective cation current. Our findings suggest that the latter relatively larger current masks the underlying I_CRAC_ since suppression of TRPC1 function either by shTRPC1 or by expression of the STIM1(^684^EE^685^) mutant, which does not gate TRPC1, facilitates detection of I_CRAC_. Further, SOAR-activated I_CRAC_ required Orai1 but not TRPC1. Thus Orai1 and TRPC1 are components of two distinct channels. These findings provide strong argument against the possibility that TRPC1 and Orai1 contribute to the same channel pore or that Orai1 is a regulatory, non-conducting, subunit of TRPC channels [59].

We also report that Orai1-mediated Ca^2+^ entry triggers plasma membrane recruitment of TRPC1. These data reveal a novel function for Orai1 that can explain its critical requirement in the activation of TRPC1 channels following Ca^2+^ store depletion. We show that Ca^2+^ store depletion leads to enhanced surface expression of TRPC1, which is blocked when Ca^2+^ is removed from the external medium or SOCE is inhibited by addition of Gd^3+^. Knockdown of endogenous Orai1 expression or expression of non-functional Orai1 mutants (Orai1-E106Q) also lead to loss of TRPC1 in the plasma membrane. Notably, in cells expressing Orai1-E106D, TRPC1 trafficking is supported in Ca^2+^-containing medium but not Ca^2+^-free medium. Together, these findings provide strong evidence that surface expression of TRPC1 is determined by the Ca^2+^ permeability of Orai1 and that TRPC1 is gated by STIM1 and not directly by [Ca^2+^]_i_ increase. Presently we cannot conclusively rule out the involvement of possible downstream signaling pathway(s) activated by Orai1-mediated Ca^2+^ entry.

**Figure 7:**
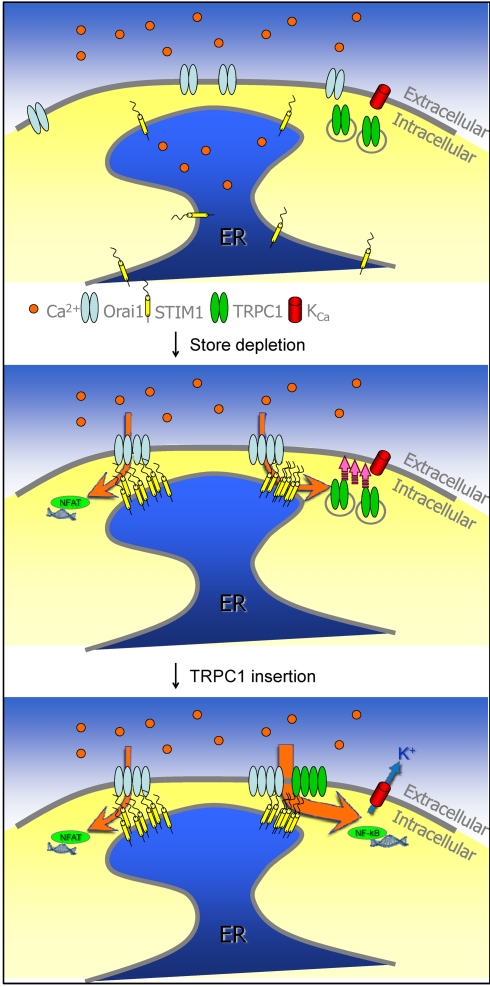
Proposed model for TRPC1 activation. In resting cells Orai1 and STIM1 have diffused localization in the PM and ER membrane, respectively. We predict that TRPC1 is localized in recycling vesicles (top panel). Following Ca^2+^ store depletion, STIM1 aggregates and translocates to the ER/PM junctional domains. Orai1 is recruited to the STIM1 puncta resulting in CRAC channel activation. The resulting [Ca^2+^]_i_ increase leads to activation of NFAT and insertion of TRPC1-vesicles into the plasma membrane (middle panel). TRPC1 is then gated by STIM1 resulting in enhancement of Ca^2+^ entry, higher [Ca^2+^]_i_, and activation of K_Ca_ channels and NFκB (lower panel).

The data presented above also reveal important aspects of TRPC1, Orai1, and STIM1 clustering that are critical in the regulation of TRPC1 within the same ER/PM junctional domains where Orai1 is regulated by STIM1. We show that in response to store depletion TRPC1 co-clusters with STIM1 and Orai1. More importantly while Orai1 is not required for clustering and association of TRPC1-STIM1, localization of STIM1 in the ER/PM junctional domains is critical for recruitment and association of Orai1 and TRPC1. Thus far there are no data to show that TRPC1 and Orai1 directly interact with each other, although both channels interact with STIM1. STIM1 interacts with Orai1 via the SOAR domain, which also leads to gating of the channel. In the case of TRPC1 while the C-terminal ^684^KK^685^ residues of STIM1 are involved in gating the channel, the ERM domain [46] could interact with the channel and serve as a scaffold to retain TRPC1 within the ER/PM junctional regions. We suggest that interaction with STIM1 allows the channels to be localized in close proximity to each other, facilitating Orai1-mediated Ca^2+^ entry to locally regulate plasma membrane insertion of TRPC1. However, our data show that internalization of TRPC1 is apparently not dependent on [Ca^2+^]_i_ (Figure 5C). Thus, TRPC1 can remain active provided the Ca^2+^ stores are depleted and STIM1 is localized in the peripheral domains. Based on our data, we suggest the following sequence of events in the activation of TRPC1: (i) Ca^2+^ store depletion leads to translocation of STIM1 to ER/PM junctional domains and recruitment of Orai1 (localized within the plasma membrane) and TRPC1 (likely localized in intracellular trafficking vesicles), (ii) Orai1 is activated by STIM1 and Ca^2+^ entry via Orai1 triggers exocytosis of TRPC1, and finally (iii) STIM1 gates plasma membrane TRPC1 (depicted in the model shown in Figure 7).

We also demonstrate the unique contributions of TRPC1 and Orai1 to SOCE. Remarkably, different cellular functions are regulated when Orai1 alone is activated compared to conditions when both channels are activated. Our data suggest that TRPC1 augments the [Ca^2+^]_i_ increase resulting from Orai1-mediated Ca^2+^ entry. Consistent with this, TRPC1-mediated Ca^2+^ entry is required for K_Ca_ function and contributes to NFκB activation, both of which require relatively higher [Ca^2+^]_i_, but not for NFAT activation, which can be activated at lower [Ca^2+^]_i_ (see Figure 7) [29],[60]. Interestingly, the requirement of TRPC1 for K_Ca_ activity is similar to our previous finding that submandibular gland acinar cells from TRPC1−/− mice display loss of sustained K_Ca_ activity, which accounts for the decrease in fluid secretion in these glands. We have previously shown that TRPC1 is localized in the basal and lateral regions of acinar cells [29],[52] and that TRPC1 and STIM1 associate following stimulation of acini [53]. Since Orai1 is critical for TRPC1 function, localization of these proteins in the salivary gland acinar cells is a key determinant for the functional interaction between them. Feasibility for the interaction of the three proteins and regulation of TRPC1 in the gland is demonstrated by our data (Figure S6), showing that following agonist stimulation Orai1, TRPC1, and STIM1 are strongly co-localized in the lateral membrane region of acinar cells while TRPC1 and STIM1 also appear to be colocalized in the basal region. In salivary gland acinar cells agonist stimulation leads to [Ca^2+^]_i_ elevation, which is first detected in the apical region of the cells and then spreads to basal and lateral regions, irrespective of the level of stimulation [55],[61]. Although further studies will be required to confirm the presence of Orai1 in the basal membrane region of acini, co-localization of TRPC1, Orai1, and STIM1 in the lateral membrane region of stimulated cells supports our suggestion that Orai1 can regulate TRPC1 function in this region and thus modulate SOCE.

In conclusion, the data described above reveal novel insight into the molecular components and regulation of TRPC1-SOC channels. Our findings provide strong evidence that TRPC1 and Orai1 constitute distinct SOC and CRAC channels, respectively, both of which are gated by STIM1 in response to store depletion and contribute to SOCE in the same cell. The critical step in the activation of TRPC1 is its insertion into the plasma membrane, which is governed by Orai1-mediated local Ca^2+^ entry. In addition to gating TRPC1 and Orai1, STIM1 also mediates the association of the two channels within discrete ER/PM junctional domains, which is the site for SOCE [19],[21]. The three proteins are also co-localized in the membrane region predicted to be the site of SOCE in acinar cells [56],[57], thus highlighting the potential physiological relevance of our findings. Importantly, TRPC1 augments Ca^2+^ entry mediated by Orai1-CRAC channels and is required for activation of K_Ca_ channels and NFκB, but not NFAT, signaling. As has been suggested, the amplitude, frequency of oscillations, or spatial patterning of [Ca^2+^]_i_ changes determines the regulation of different cell functions [1],[4],[51],[55],[60],[62]. Although further studies are required to elucidate exactly how TRPC1 alters the primary [Ca^2+^]_i_ signals generated by Orai1, the present data suggest that regulation of TRPC1 trafficking can provide a mechanism for rapidly modulating [Ca^2+^]_i_. STIM1 is emerging as a versatile ER Ca^2+^ sensor that regulates multiple target proteins in response to Ca^2+^ store depletion. In addition to activation of Orai1 and TRPC channels, STIM1 has been reported to inhibit Cav1.2 channels [63],[64] and activate adenylyl cyclase [65], both of which depend on Ca^2+^ store depletion. While regulation of TRPC1 and Cav1.2 require association of the channels with Orai1 within ER/PM junctional domains, Orai1 function does not appear to be involved in STIM1-dependent inhibition of Cav1.2. Thus Orai1 and STIM1 by coordinating the regulation of other ion channels and signaling components can modulate [Ca^2+^]_i_ and critically impact SOCE-mediated Ca^2+^ signaling and a variety of cellular functions.

## Methods

### Cell Culture, RNAi Transfection, and Reagents

HSG cells were cultured in MEM medium, supplemented with 10% heat-inactivated fetal bovine serum, and 1% penicillin/streptomycin. Sequences for the siOrai1, siSTIM1, and shTRPC1 targeting to human Orai1, STIM1, and TRPC1, respectively, were similar to previously described sequences [42]. All siRNA duplexes were obtained from Dharmacon. Lipofectamine RNAiMAX (Invitrogen) was used for siRNA transfection while Lipofectamine 2000 was used for other plasmids. Cells were typically transfected 24 h after plating and experiments were performed 48 h post-transfection. All other reagents used were of molecular biology grade obtained from Sigma Aldrich unless mentioned otherwise.

### [Ca^2+^]_i_ Measurements

Fura-2 fluorescence was measured in single HSG cells cultured for 24 h in glass bottom MatTek tissue culture dishes (MatTek Corp. Ashland, MA) and transfected as required; experiments were done 48 h post-transfection. Cells were loaded with 5 µM Fura-2 (Invitrogen) for 30 min at 37°C. Fluorescence was recorded using a Till Photonics-Polychrome V spectrofluorimeter and MetaFluor imaging software (Molecular Devices). Each fluorescence trace (340/380 nm ratio) represents an average from at least 50–150 cells from >6 individual experiments. Student's *t* test was used to statistically evaluate the data.

### Electrophysiology

Coverslips with HSG cells were transferred to the recording chamber and perfused with Ca^2+^ containing standard external solution (Ca^2+^-SES) with the following composition (in mM): NaCl, 145; KCl, 5; MgCl_2_, 1; CaCl_2_, 1; Hepes, 10; glucose, 10; pH 7.4 (NaOH). The patch pipette had resistances between 3 and 5 milliohms after filling with the standard intracellular solution that contained the following (in mM): cesium methane sulfonate, 145; NaCl, 8; MgCl_2_, 10; Hepes, 10; EGTA, 10; pH 7.2 (CsOH). For K_Ca_ measurements, pipette solution contained 150 mM KCl, 2 mM MgCl_2_, 1 mM Mg-ATP, 5 mM Hepes, 0.1 mM EGTA, and pH 7.2, potassium hydroxide. Osmolarity for all the solutions was adjusted with mannose to 300±5 mosM using a vapor pressure Osmometer (Wescor, Logan, UT). All electrophysiological experiments were performed in the tight-seal whole cell configuration at room temperature (22–25°C) using an Axopatch 200B amplifier (Molecular Devices). Development of the current was assessed by measuring the current amplitudes at a potential of −80 mV, taken from high resolution currents in response to voltage ramps ranging from −90 to 90 mV over a period of 100 ms imposed every 2 s (holding potential was 0 mV) and digitized at a rate of 1 kHz. Liquid-junction potentials were less than 8 mV and were not corrected. Capacitative currents and series resistance were determined and minimized. For analysis, the current recorded during the first ramp was used for leak subtraction of the subsequent current records. Thapsigargin (Tg 1 µM), dissolved in the bath solution, was used to stimulate the cells. DVF solution contains (mM): NaCl 165; CsCl 5; EDTA 10; HEPES 10; glucose 10; pH 7.4 (NaOH). Cells were pretreated with 1 µM Tg for 10 min either in Ca^2+^ containing or Ca^2+^ free medium before whole cell configuration was achieved. Cells were switched to DVF 1 min after achieving whole cell configuration in Ca^2+^ free external medium.

### Immunoprecipitation and Western Blotting

Transfected HSG cells were washed with phosphate-buffered saline (PBS) and lysed in radioimmunoprecipitation assay (RIPA) protein extraction buffer (50 mM Tris-HCl, 150 mM NaCl, 0.1% sodium dodecyl sulfate (SDS), 0.5% sodium deoxycholate, 1% Triton X-100, 2 mM EDTA, 1 mM dithiothreitol (DTT), pH 7.4) supplemented with Complete Protease Inhibitor Cocktail tablets (Roche Diagnostics). Where indicated, cells were first stimulated for 5 min with 1 µM Thapsigargin (Tg), lysates was then centrifuged at 12,000 x g for 30 min at 4°C, and the supernatant was collected and analyzed by SDS-PAGE and Western blotting (50 µg of protein were loaded per lane). Protein concentrations in the lysate was adjusted to 2 mg/ml and incubated with 10 µg/ml IP antibody. Immunoprecipitates were released by incubating in SDS-sample buffer and resolved in 4%–12% NuPAGE gels (Invitrogen) followed by Western blotting. Anti-STIM1 (Cell signaling technology, Danvers, MA), anti-Orai1 (Open Biosystems, Huntsville, AL), anti-GAPDH (Abcam Inc, Cambridge, MA), and Anti-TRPC1 antibody [42] were used at 1∶1000, 1∶1000, 1∶10000, and 1∶400 dilution, respectively.

### Cell Surface Biotinylation

Cells were transfected with vector or scrambled control as required. For stimulation experiments, cells were pretreated with 1 µM Thapsigargin in the presence (+Ca^2+^) or absence (−Ca^2+^) of extracellular calcium, and incubation time was 5 min or otherwise as indicated. The reaction was stopped by adding ice-cold quenching solution. In BAPTA-AM loading experiments, cells were pretreated with 200 µM BAPTA-AM (Invitrogen) in SES containing 100 µM extracellular Ca^2+^ for 30 min at 37°C. Treated cells were then incubated for 20 min with 1.5 mg/ml Sulfo-NHS-LC-Biotin (Pierce) in 1XPBS (pH 8.0) on ice. Following biotin labeling, cells were washed and harvested in RIPA buffer using the same protocol as described above. Biotinylated proteins were pulled down with NeutrAvidin-linked beads (Pierce) and detected by Western blotting. Band intensities of surface proteins were obtained using Image J software.

### Luciferase Reporter Assays

NFκB-Luc, NFAT-Luc, and hRLuc-TK were obtained from Promega. HSG cells were transfected with the indicated constructs with either NFκB or NFAT reporter gene, and co-transfection with the Renilla luciferase gene (hRLuc-TK) driven by the TK promoter was used to control for cell number and transfection efficiency. Transfected cells were stimulated as described in [60]. Luciferase activity was measured with the Dual-Glo Luciferase Assay System (Promega). For each condition, luciferase activity was measured with four samples taken from duplicate wells with a 96-well automated luminometer (Turner Biosystems). Results are represented as the ratio of firefly to Renilla luciferase activity.

### TIRF Microscopy

An Olympus IX81 motorized inverted microscope (Olympus) was used as described previously [42] using 447, 514, and 568 nm lasers for excitation of CFP, YFP, and mCherry, respectively, and a TIRF-optimized Olympus Plan APO 60x (1.45 NA) oil immersion objective and Lambda 10-3 filter wheel (Sutter Instruments) containing 480-band pass (BP 40 m), 525-band pass (BP 50 m), and 605-band pass (BP 52 m) filters for emission. Images were collected using a Hamamatsu EM C9100 camera (Hamamatsu) and the MetaMorph imaging software (Molecular Devices). MetaMorph was also used to measure the fluorescence intensity before and after stimulation with Tg. Briefly, regions of interest were selected to obtain the values for their fluorescence intensities during a time course experiment. These values were then plotted using the Origin 8 software (OriginLab).

### Immunofluorescence

Balb/c mice were anesthetized and injected subcutaneously with either saline (Resting) or 0.5 mg of pilocarpine/kg (Stimulated). After the saliva secretion was observed in stimulated mice, the animals were perfusion fixed with 10% buffered formalin and immediately euthanized. Salivary glands were excised and embedded in paraffin for histologic processing. Slides of paraffin sections were deparaffinized and rehydrated. Sections were unmasked by microwaving samples for 10 min in a microwave pressure cooker (NordicWare) in 1 mM EDTA, pH 8.0, containing 0.05% Tween 20. After cooling, sections were blocked either with 0.5% BSA in PBS (for direct conjugates) or with 10% donkey serum in PBS (for samples using secondary antibodies). After blocking for 30 min at room temperature, primary antibodies were applied and incubated at 4°C overnight. For samples using two or more rabbit host primary antibodies, direct-conjugation with a fluorescent tag using Invitrogen's Zenon labeling kit was used. For antibodies requiring secondary antibody labeling, donkey anti-rabbit Alexa conjugates were used (Invitrogen). A negative control using normal rabbit IgG at the same concentration as specific primaries was included for both methods. After labeling with primary antibodies only, samples were washed extensively and incubated with secondary antibodies for 1 h at room temperature, washed, and mounted with VectaShield mounting medium containing DAPI. Zenon conjugated samples were washed extensively and mounted with cover slips as above. Images were collected by using a Leica Confocal microscope and MetaMorph software (Molecular Devices, Sunnyvale, CA).

### Statistics

Data analysis was performed using Origin 8 (OriginLab). Statistical comparisons were made using student's *t* test. Experimental values are expressed as means ± SD or SEM. Differences in the mean values were considered to be significant at *p*<0.01.

## Supporting Information

Figure S1 (A) Western blot showing knockdown of TRPC1 in HSG cells treated with shTRPC1 for 48 h. 5 µg of the construct was used for transfection in all the experiments shown in the article. (B–G) Effect of 1 µM Gd^3+^ and 20 µM 2APB on endogenous SOCE as well as in cells expressing TRPC1, STIM1, and Orai1 (as indicated in the figure). 1 µM Gd^3+^ or 20 µM 2APB were added immediately before Ca^2+^. (G) SOCE measured under the different conditions. ** indicates values that are significantly different from that in control cells (*p*<0.01, *n* = 50–60 cells per condition). Ca^2+^ entry under all the different conditions is blocked by 1 µM Gd^3+^ or 20 µM 2APB.
(TIF)

Figure S2 Contribution of TRPC1, STIM1, and Orai1 to Ca^2+^ entry stimulated by maximal and sub-maximal stimulation of HSG cells with CCh. Cells were transfected with shTRPC1, siOrai1, or siSTIM1 for 48 h. Cells were treated with CCh; 100 µM (A, maximal stimulation) or 1 µM (B, submaximal stimulation) in Ca^2+^ free medium for 5 min (release component not shown) prior to addition of Ca^2+^. Average values for Ca^2+^ entry are shown in (C). ** indicates cells significantly different from the control conditions (*p*<0.01, *n* = 50–80 cells from four experiments). The relative contributions of the three proteins in the two conditions of stimulation are similar.
(TIF)

Figure S3 STIM1(^684^EE^685^) activates Orai1 but not TRPC1-mediated SOCE. (A) HEK293 cells were transfected with TRPC1, STIM1, STIM1(^684^EE^685^), or STIM1(^684^EE^685^) + TRPC1(^639^KK^640^). (B) HEK293 cells were transfected with Orai1, STIM1, or STIM1(^684^EE^685^). Additions of Tg or Ca^2+^ are indicated. (C) Average data showing statistical analysis. ** indicates values significantly different from the control value in each case (*p*<0.01, *n* = 30–60 cells from three experiments).
(TIF)

Figure S4 Co-localization of STIM1 and TRPC1 is stimulated by store depletion. (A) Left: TIRFM image of HSG cells expressing CFP-STIM1 and YFP-TRPC1 (CFP-STIM1, red, and YFP-TRPC1, green; yellow clusters show co-localization of the proteins in ER/PM junctional domains). Right, upper panel shows five cells used to evaluate the increase in fluorescence intensity of the two proteins; 5 was a control ROI shown in the graph given below. Accumulation of both proteins increased at the same rate (each has been expressed relative to their respective maximum fluorescence). (B) Similar experiment using cells in which Orai1 had been knocked down by siOrai1 (Cherry-STIM1, red, and YFP-TRPC1, green). Other details are similar to that given for (A). Knockdown of Orai1 does not affect STIM1-TRPC1 clustering stimulated by Ca^2+^ store depletion.
(TIF)

Figure S5 Effect of including Ca^2+^ buffers in the pipette solution on Tg-stimulated currents. (A) [Ca^2+^]_i_ was clamped in the pipette solution at the levels indicated. I_SOC_ was not activated by increasing the [Ca^2+^] in the pipette solution to 1 µM but was activated following Tg stimulation of cells with pipette solution buffered to 100 nM Ca^2+^. (B, C) Inclusion of 10 mM BAPTA instead of 10 mM EGTA in the pipette solution did not change the activation and properties of Tg-stimulated I_SOC_ (development of current at −80 mV is shown in left panel, I–V relationships of respective currents are shown in right panel). (D) K_Ca_ channel activity measured in Tg-stimulated HSG cells with 10 mM BAPTA in the pipette solution, and Ca^2+^ in external solution was increased from 1 mM to 10 mM (third panel in C).
(TIF)

Figure S6 Localization of Orai1, TRPC1, STIM1, and AQP5 in resting and pilocarpine stimulated submandibular glands. Salivary gland sections were obtained from mice (treated as described) and used for immunofluorescence (conditions used for sample processing and labeling are provided in Materials and Methods). (A) Localization of Orai1 (left panels, green) and AQP5+Orai1 (right panels, same section as those shown in the left, AQP5 indicated in red) in resting (upper panels) and stimulated (lower panels) samples. (B) Localization of TRPC1 (left panels, green) and STIM1 (red signal, right panels, same section as those shown in the left) in resting (upper panels) and stimulated (lower panels) samples. Anti-TRPC1 (as described in Materials and Methods), anti-STIM1 and anti-Orai1 (kindly provided by Dr. Stefan Feske, New York University), and anti-AQP5 (Alomone Labs) were used.
(TIF)

## Abbreviations

CRAC: Ca^2+^ release-activated Ca^2+^
SOAR: STIM1-Orai1-activating region
SOCE: store-operated Ca^2+^ entry
