# Supplementary figures and images for: Local Ca2+ Entry Via Orai1 Regulates Plasma Membrane Recruitment of TRPC1 and Controls Cytosolic Ca2+ Signals Required for Specific Cell Functions
Source: PLoS Biol. 2011 Mar 8;9(3):e1001025. doi: 10.1371/journal.pbio.1001025 (PMC3050638; doi:10.1371/journal.pbio.1001025)

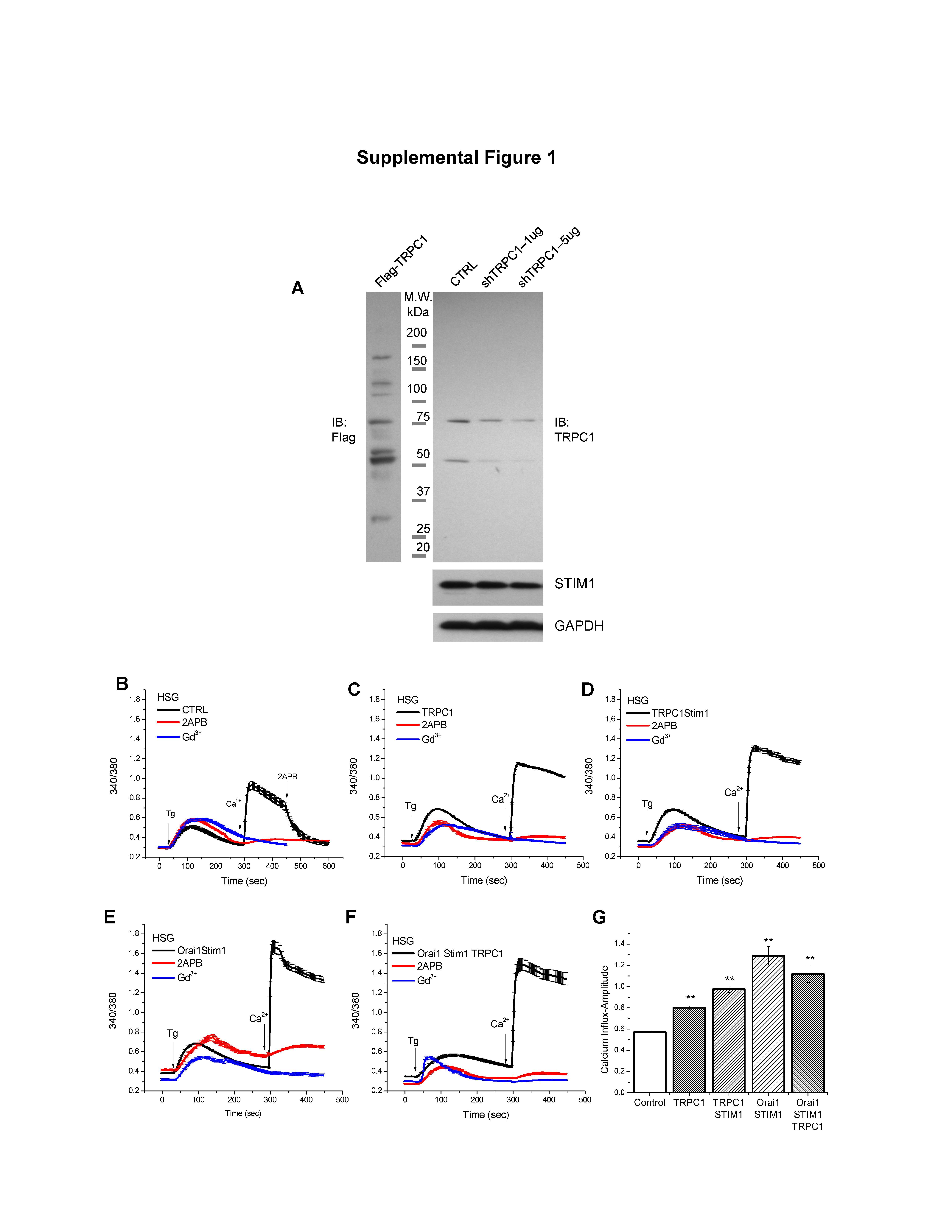

Supplement: Figure S1 — (A) Western blot showing knockdown of TRPC1 in HSG cells treated with shTRPC1 for 48 h. 5 µg of the construct was used for transfection in all the experiments shown in the article. (B–G) Effect of 1 µM Gd3+ and 20 µM 2APB on endogenous SOCE as well as in cells expressing TRPC1, STIM1, and Orai1 (as indicated in the figure). 1 µM Gd3+ or 20 µM 2APB were added immediately before Ca2+. (G) SOCE measured under the different conditions. ** indicates values that are significantly different from that in control cells (p<0.01, n = 50–60 cells per condition). Ca2+ entry under all the different conditions is blocked by 1 µM Gd3+ or 20 µM 2APB. (TIF) [file pbio.1001025.s001.tif]

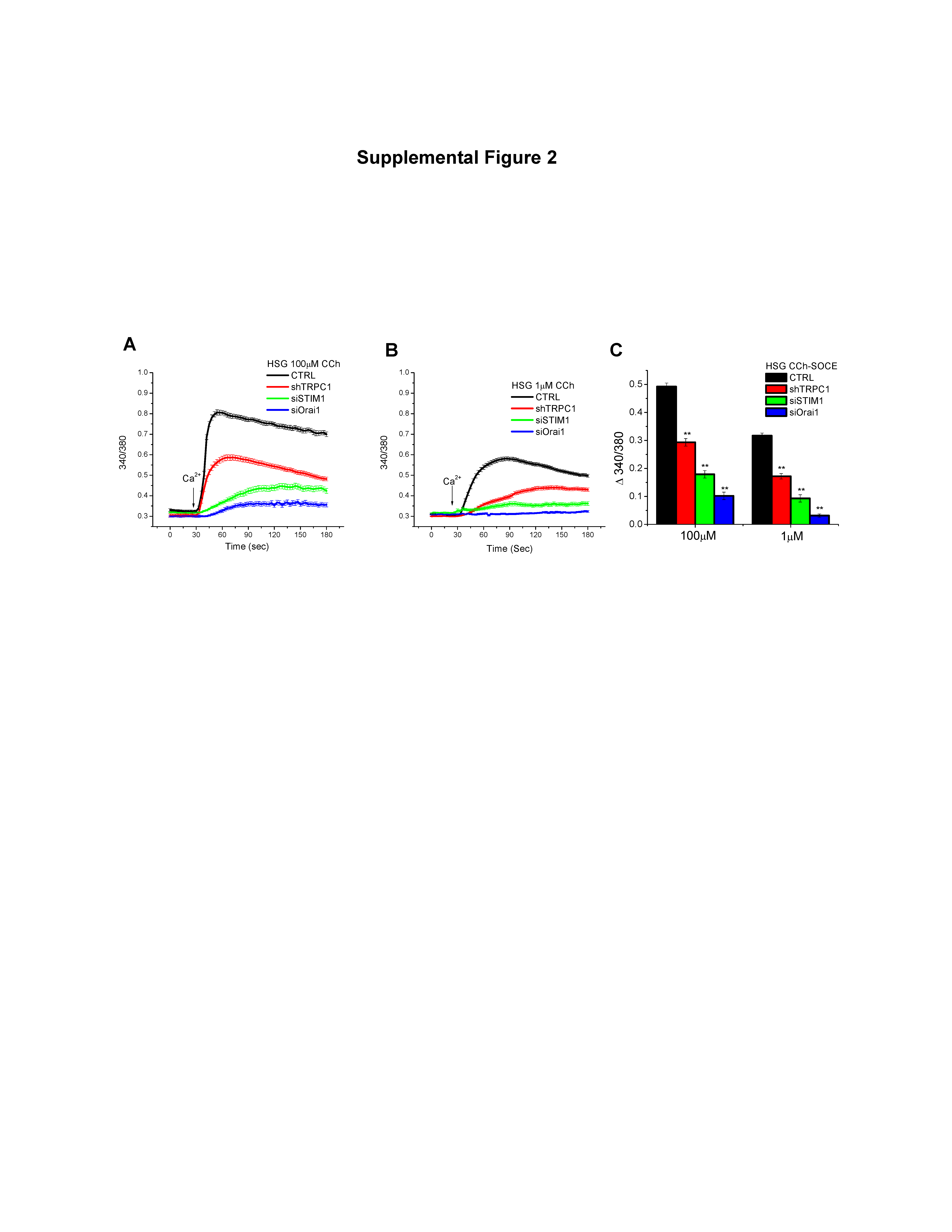

Supplement: Figure S2 — Contribution of TRPC1, STIM1, and Orai1 to Ca2+ entry stimulated by maximal and sub-maximal stimulation of HSG cells with CCh. Cells were transfected with shTRPC1, siOrai1, or siSTIM1 for 48 h. Cells were treated with CCh; 100 µM (A, maximal stimulation) or 1 µM (B, submaximal stimulation) in Ca2+ free medium for 5 min (release component not shown) prior to addition of Ca2+. Average values for Ca2+ entry are shown in (C). ** indicates cells significantly different from the control conditions (p<0.01, n = 50–80 cells from four experiments). The relative contributions of the three proteins in the two conditions of stimulation are similar. (TIF) [file pbio.1001025.s002.tif]

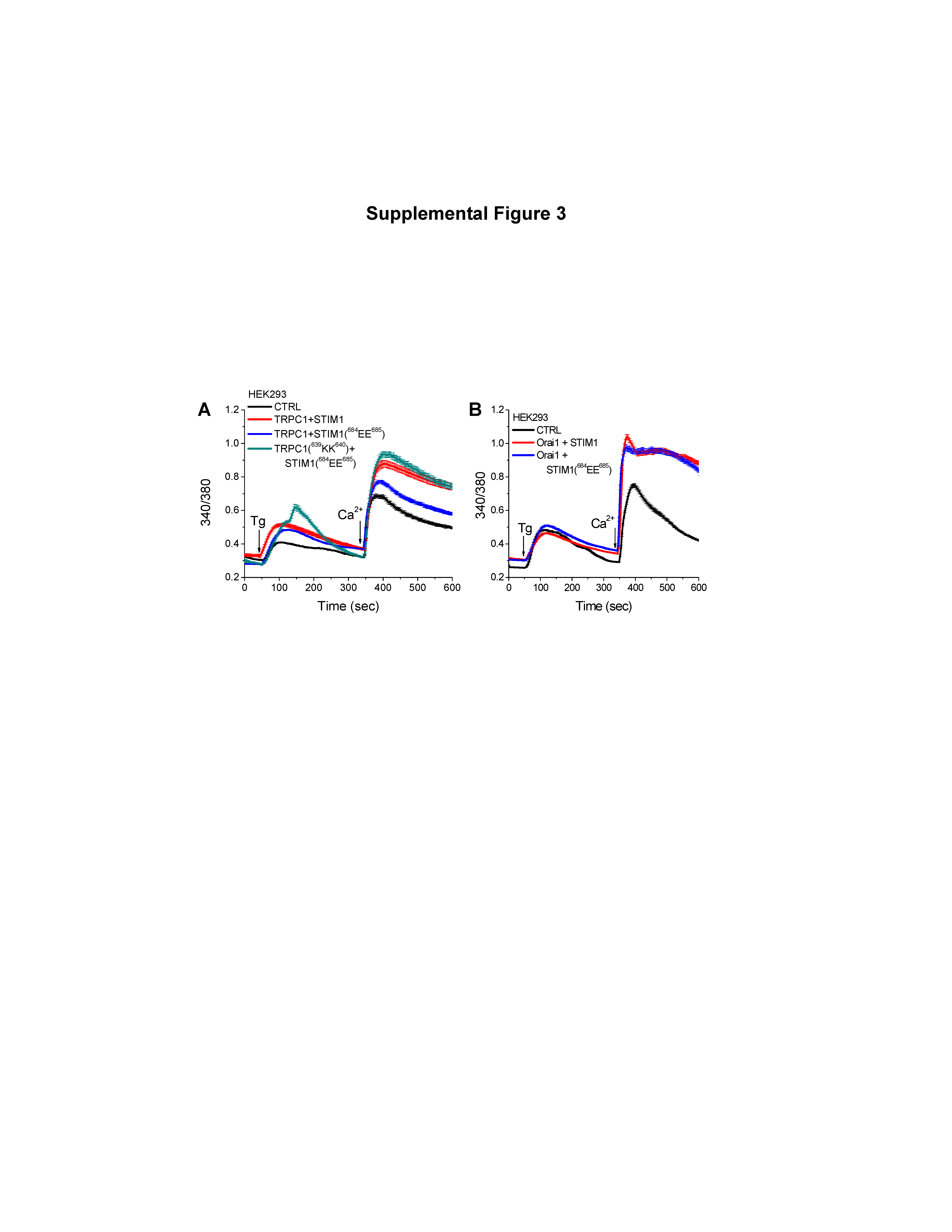

Supplement: Figure S3 — STIM1(684EE685) activates Orai1 but not TRPC1-mediated SOCE. (A) HEK293 cells were transfected with TRPC1, STIM1, STIM1(684EE685), or STIM1(684EE685) + TRPC1(639KK640). (B) HEK293 cells were transfected with Orai1, STIM1, or STIM1(684EE685). Additions of Tg or Ca2+ are indicated. (C) Average data showing statistical analysis. ** indicates values significantly different from the control value in each case (p<0.01, n = 30–60 cells from three experiments). (TIF) [file pbio.1001025.s003.tif]

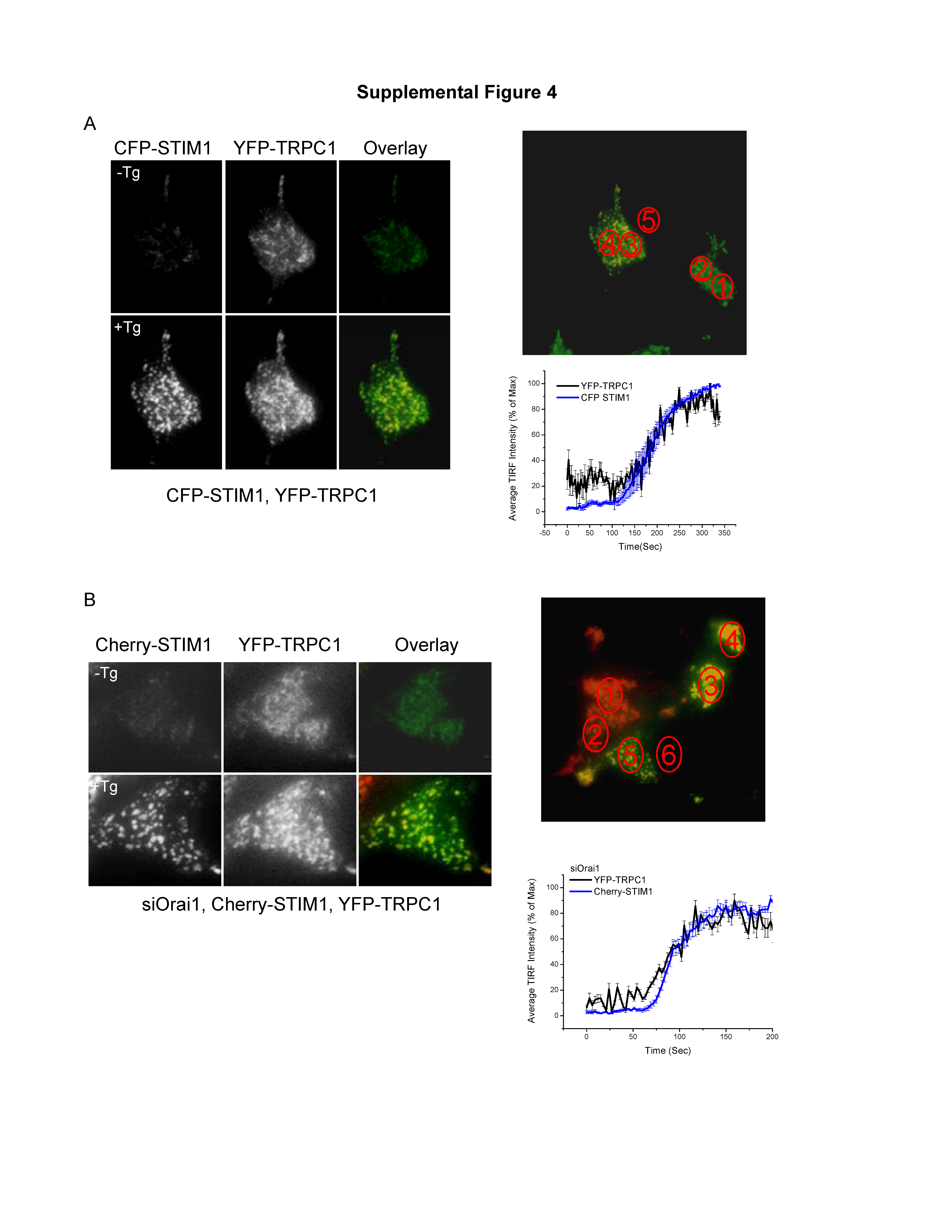

Supplement: Figure S4 — Co-localization of STIM1 and TRPC1 is stimulated by store depletion. (A) Left: TIRFM image of HSG cells expressing CFP-STIM1 and YFP-TRPC1 (CFP-STIM1, red, and YFP-TRPC1, green; yellow clusters show co-localization of the proteins in ER/PM junctional domains). Right, upper panel shows five cells used to evaluate the increase in fluorescence intensity of the two proteins; 5 was a control ROI shown in the graph given below. Accumulation of both proteins increased at the same rate (each has been expressed relative to their respective maximum fluorescence). (B) Similar experiment using cells in which Orai1 had been knocked down by siOrai1 (Cherry-STIM1, red, and YFP-TRPC1, green). Other details are similar to that given for (A). Knockdown of Orai1 does not affect STIM1-TRPC1 clustering stimulated by Ca2+ store depletion. (TIF) [file pbio.1001025.s004.tif]

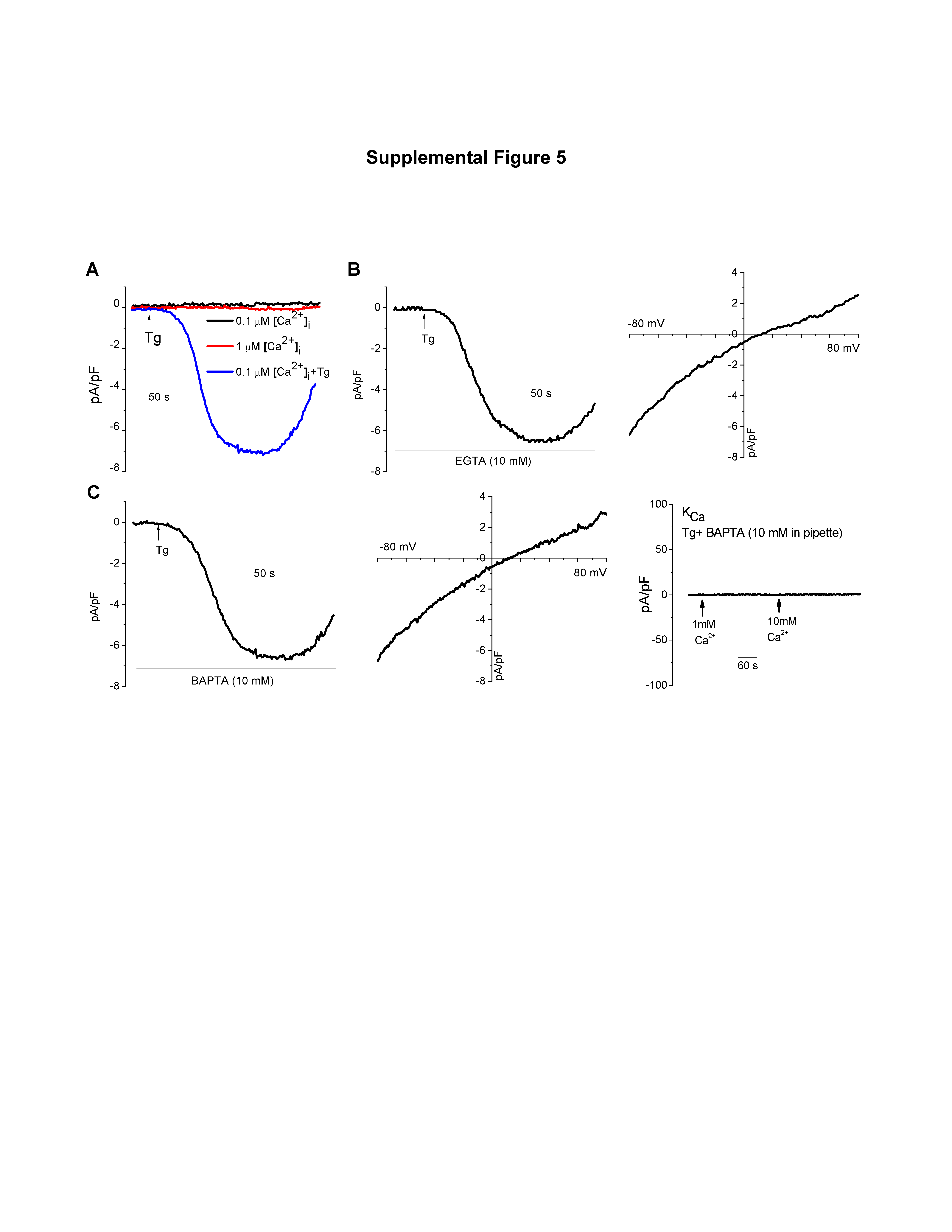

Supplement: Figure S5 — Effect of including Ca2+ buffers in the pipette solution on Tg-stimulated currents. (A) [Ca2+]i was clamped in the pipette solution at the levels indicated. ISOC was not activated by increasing the [Ca2+] in the pipette solution to 1 µM but was activated following Tg stimulation of cells with pipette solution buffered to 100 nM Ca2+. (B, C) Inclusion of 10 mM BAPTA instead of 10 mM EGTA in the pipette solution did not change the activation and properties of Tg-stimulated ISOC (development of current at −80 mV is shown in left panel, I–V relationships of respective currents are shown in right panel). (D) KCa channel activity measured in Tg-stimulated HSG cells with 10 mM BAPTA in the pipette solution, and Ca2+ in external solution was increased from 1 mM to 10 mM (third panel in C). (TIF) [file pbio.1001025.s005.tif]

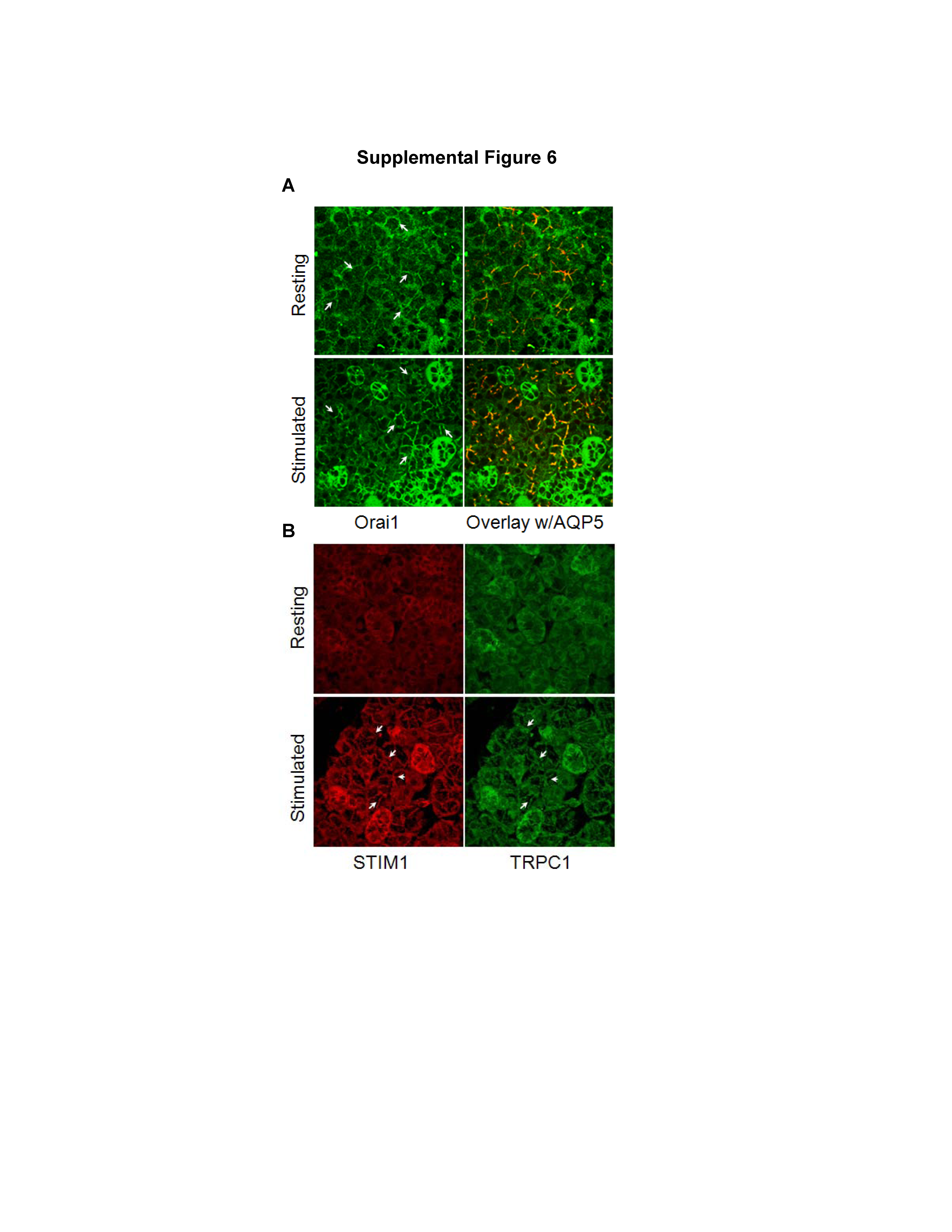

Supplement: Figure S6 — Localization of Orai1, TRPC1, STIM1, and AQP5 in resting and pilocarpine stimulated submandibular glands. Salivary gland sections were obtained from mice (treated as described) and used for immunofluorescence (conditions used for sample processing and labeling are provided in Materials and Methods). (A) Localization of Orai1 (left panels, green) and AQP5+Orai1 (right panels, same section as those shown in the left, AQP5 indicated in red) in resting (upper panels) and stimulated (lower panels) samples. (B) Localization of TRPC1 (left panels, green) and STIM1 (red signal, right panels, same section as those shown in the left) in resting (upper panels) and stimulated (lower panels) samples. Anti-TRPC1 (as described in Materials and Methods), anti-STIM1 and anti-Orai1 (kindly provided by Dr. Stefan Feske, New York University), and anti-AQP5 (Alomone Labs) were used. (TIF) [file pbio.1001025.s006.tif]
